# Gastrogastric intussusception in adults: a case report with review of the literature

**DOI:** 10.1259/bjrcr.20180006

**Published:** 2018-07-10

**Authors:** Arash Behrooz, Morgan Cleasby

**Affiliations:** 1 Department of Pathology, Worcestershire Royal Hospital, Worcester, UK; 2 Department of Radiology, Good Hope Hospital, Birmingham, UK

## Abstract

Intussusception of the gastrointestinal viscera is rarely encountered in adult patients and is frequently associated with a polypoidal lead point, which is often malignant. We would like to present the case of a 68-year-old male with a history of decompensated liver disease and multiple medical comorbidities, who was discovered to have an incidental gastrogastric intussusception on CT. No polypoidal lead point was seen and we believe this to be the first case of its kind to be described. We suggest that distortions in the patient’s visceral and vascular anatomy and raised intra-abdominal pressure resulting from concomitant ascites, hiatus hernia, portal hypertension and oesophageal varices have provided an alternative mechanism for a gastrogastric intussusception to develop.

## Background

The earliest known description of intussusception was made by the Dutch physician Paul Barbette in 1674, with successful manual reduction of an intussusception being first performed by Sir Jonathan Hutchinson in 1871 in a 2-year-old girl following failure of hydrostatic reduction.^1^ The role of radiographic contrast enema in diagnosing colonic intussusception was first outlined by William E Ladd in 1913 with Retan and Stephens carrying out the first successful contrast enema reduction in 1926.^2^

Intussusception in adult patients is encountered far less commonly than in paediatric populations, representing 5% of all intussusceptions, with an incidence of 2–3 cases per million adults per annum^3^ . Intussusception in adults most frequently occurs in the small bowel, with 10% of cases occurring in the stomach or at the site of a surgical stoma.^4^ We were unable to find epidemiological data relating to gastrogastric intussusception specifically. Unlike in children, adult intussusceptions are rarely idiopathic, with an identifiable cause observed in 70–90% of cases.^5^ Typically, lead points are malignant neoplasms (attributed to 66% of colonic intussusceptions and 30% of enteric intussusceptions), with other polypoidal lead points including stromal tumours, lipomas, hyperplastic polyps and hamartomas.^6^ Adults presenting with intussusception commonly have non-specific symptoms, *e.g.* abdominal pain and vomiting, with intestinal obstruction and an abdominal mass encountered less frequently.^7^

## Literature review

Case discussions and reviews of gastrogastric intussusception are infrequently reported in medical literature. A search of the literature was carried out using PubMed in June 2017. Data from previous studies in accessible journals with available abstracts is presented in Table 1.

**Table 1.** Case reports on gastrogastric intussusception 1950–2017

**Author**	**Age/sex**	Presentation	**Diagnosis**	Radiological features	Histological findings
Thompson ^8^	72 M	Epigastric pain, nausea, vomiting	Laparotomy	Not stated	Pedunculated intragastric tumour
Raw ^9^	66 F	Epigastric discomfort, vomiting	Laparotomy	Not stated	Malignant gastric papilloma
Grundy et al^10^	78 F and 76 F	Weight loss, dysphagia, vomiting, epigastric pain	Fluoroscopy	Fundal mass intussuscepting into antrum with pseudopedicle	Leiomyoma
Javors et al^11^	81 F	Anaemia	Single contrast UGI series	Foreshortening of stomach with pseudopedicle, antral ovoid mass, coiled spring appearance	Leiomyoma with leiomyoblastomatous elements
Vikram et al^12^	65 F	Epigastric pain, nausea, vomiting, epigastric mass	Double contrast barium meal CT abdomen	Bird’s beak appearance Invagination of wall of greater curve into gastric lumen	Hypertrophic gastric polyps
Shanbhogue et al^13^	83 F	Melaena, weight loss, anaemia	CT abdomen	Target sign	Gastrointestinal stromal tumour
Eom et al^14^	73 F	Vomiting, general weakness, sepsis	OGD CT abdomen	Polypoid mass with a vascular pedicle	Gastric adenocarcinoma
Jo et al^15^	82 F	Chest pain and vomiting	CT abdomen	Mass in body of stomach telescoping into antrum	Primary gastric lymphoma
Davila et al^16^	77 F	Fever, abdominal discomfort, left lateral abdominal mass	MR abdomen	Target sign on axial STIR pT2*	Tubulo-villous adenoma

OGD, oesophagogastroduodenscopy; STIR, short tau inversion-recovery; UGI, upper gastrointestinal.

There is a diverse range of reported radiological features associated with gastrogastric intussusception which have been described in previous case reports. Fluoroscopic studies of patients presenting with this condition have revealed a “coiled spring” appearance, whilst CT and MR studies have demonstrated the appearance of a “target sign”. Both radiological features are frequently reported in cases of distal intussusception involving the intestines and it is logical that they would also be seen in cases of gastrogastric intussusception as the mechanism of anatomical distortion which results in telescoping of all layers of the wall of the viscus is conserved.

Ultrasound features of gastrogastric intussusception have not been described, though positive ultrasound findings, *e.g.* a target sign are frequently observed in cases of intestinal intussusception. It is also noteworthy that all but one of the patients who have been included in the above case reports were female, although the evidence for a gender predominance in intussusceptions in general is equivocal.^17^

The evidence from previous studies also indicates that amongst reported cases of gastrogastric intussusception in humans, a soft tissue growth is a typical concurrent finding and lead point. This may be of a malignant or benign histological nature. However, there is a paucity of evidence relating to non-tumorous lesions which may be concomitant or which may indeed predispose individuals to gastrogastric intussusception. Interestingly, there are several case reports in veterinary journals relating to gastric intussusceptions presenting in dogs with a greater diversity of non-tumorous aetiological factors compared with humans, including dietary indiscretion and presence of a foreign body.^18^

In this case report, we would like to present a case of gastrogastric intussusception occurring in a patient without a concurrent tumour and discuss alternative pathophysiological mechanisms which may predispose individuals to this condition.

## Clinical presentation

A 68-year-old male was admitted to the acute medical unit in April 2017 complaining of acute onset left-sided abdominal pain, general weakness, dysphagia, vomiting and poor appetite. He had received inpatient care twice in the month prior to his most recent attendance and had, during his preceding admission, been diagnosed with decompensated chronic liver disease and ascites. Oesophagogastroduodenoscopy performed during a previous admission in March 2017 had revealed portal hypertensive gastritis and Grade II varices in the middle and lower thirds of the oesophagus.

His past medical history included a 4 cm hiatus hernia, myocardial infarction, congestive cardiac failure, peripheral vascular disease, atrial fibrillation, previous deep vein thrombosis, gallstones, and coeliac disease. He denied regular alcohol consumption. Laboratory investigations during his final admission revealed an acute kidney injury, deranged liver function tests and a leucocytosis. Serratia species were isolated in the ascitic fluid. Despite antibiotic therapy, the patient remained septic and died 14 days following admission. Following post-mortem examination, the cause of death was recorded as spontaneous bacterial peritonitis on a background of cirrhosis with portal hypertension.

## Imaging findings

**Figure 1.**
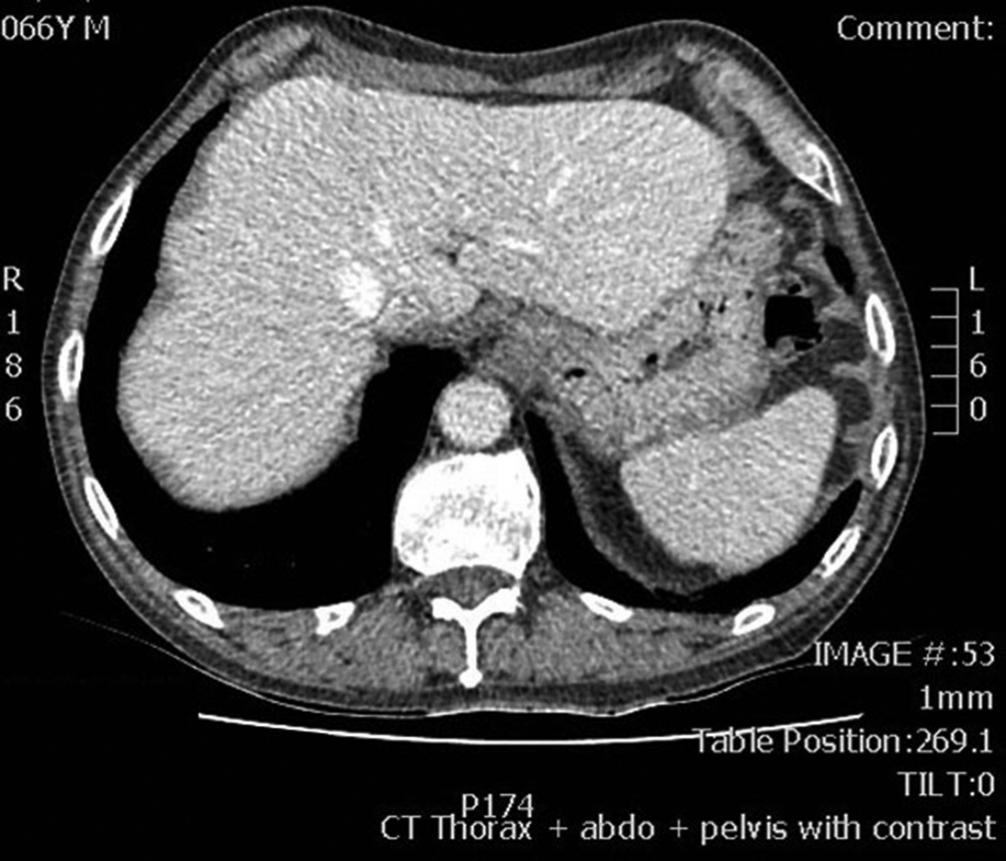
CT abdomen and pelvis January 2015 demonstrating a normal appearing stomach.

The patient had previously had a CT scan of the abdomen and pelvis on 19 January 2015, at which time a normal appearing stomach was seen without hiatus hernia (Figure 1). There was no ascites at that time.

**Figure 2.**
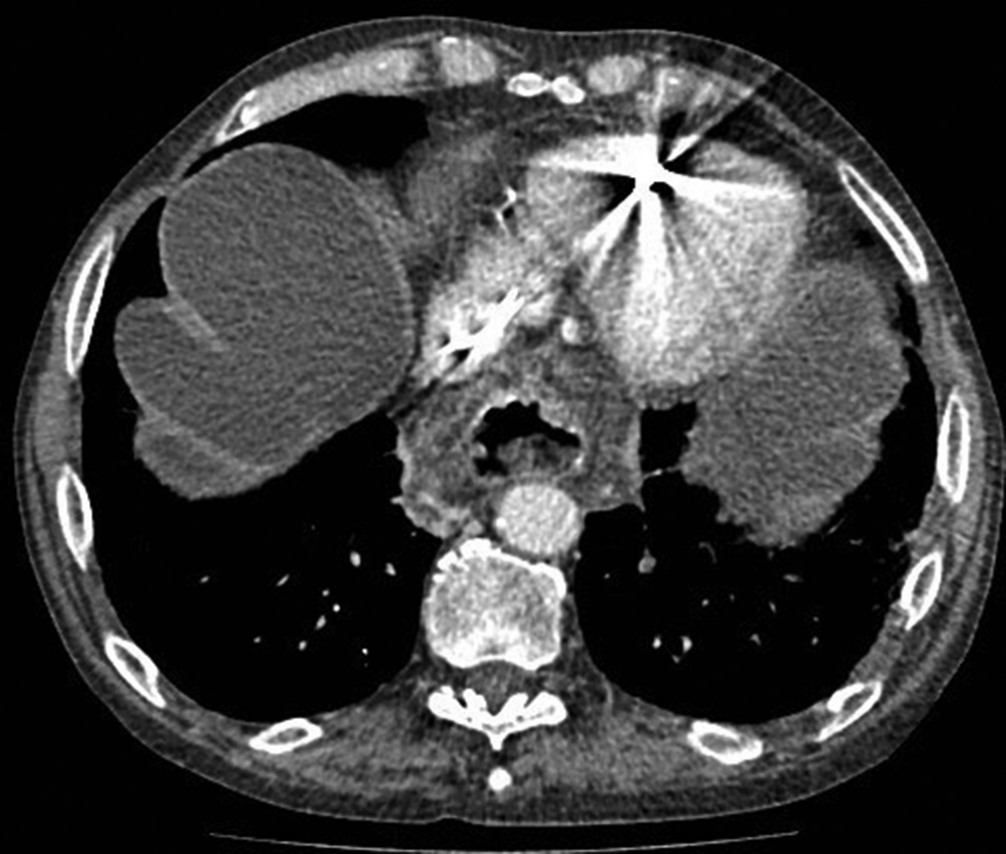
CT abdomen and pelvis March 2017 demonstrating presence of ascites and hiatus hernia.

The patient underwent a CT abdomen and pelvis study in the portal venous phase following intravenous contrast on 21 March 17—3 weeks prior to his final admission—which revealed evidence of inhomogeneous filling defects seen in the superior mesenteric vein, splenic vein and portal vein highly suggestive of partial thrombosis. The presence of a large amount of ascites was also noted and a moderately large sliding hiatus hernia was present (Figure 2).

**Figure 3.**
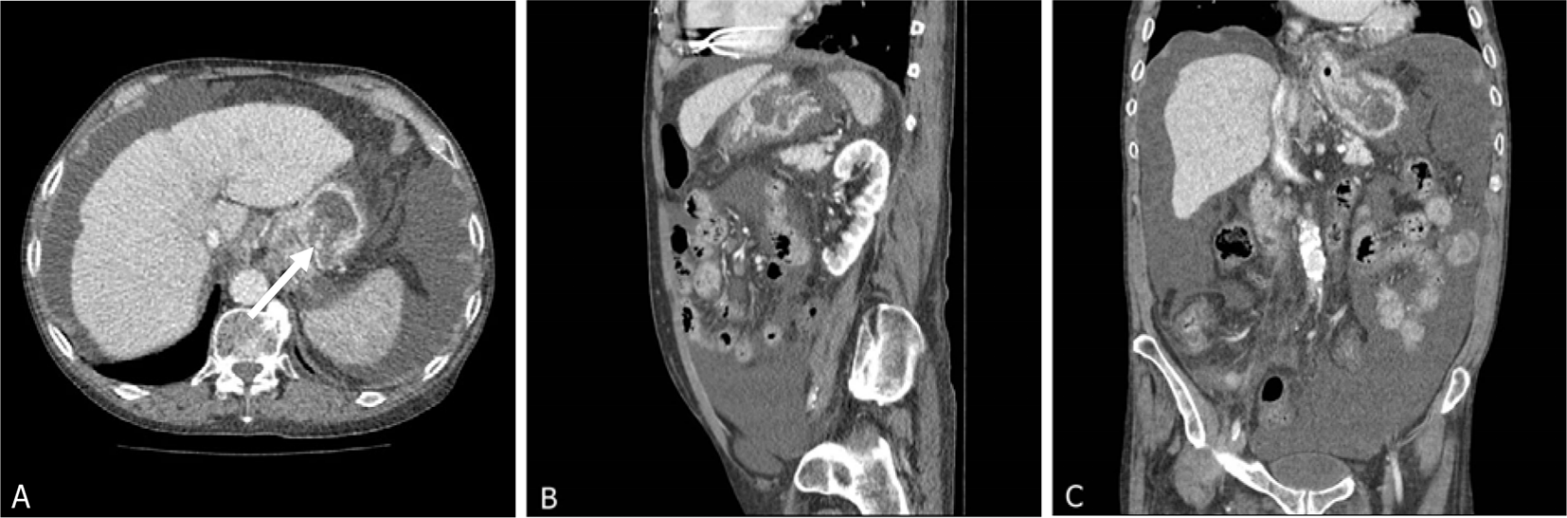
CT abdomen and pelvis March 2017 with evidence of ascites, gastrogastric intussusception and vascular pseudopedicle (white arrowhead).

In addition, a large low-density filling defect was seen within the gastric body, with a vascular pseudopedicle appearance, consistent with a gastrogastric intussusception (Figure 3A–C).

**Figure 4.**
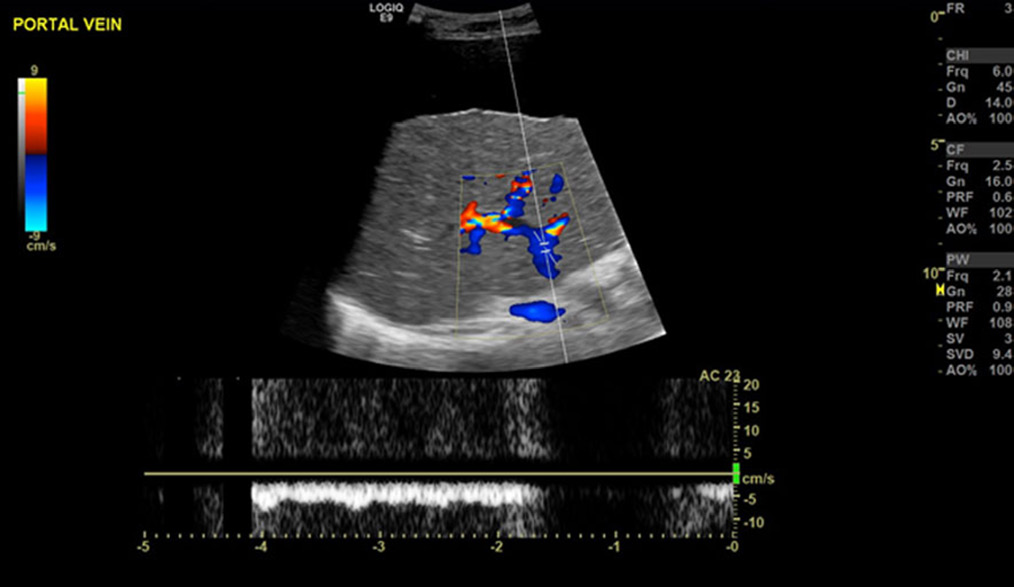
Ultrasound  abdomen April 2017 demonstrating reversal of flow in the hepatic portal vein.

During the patient’s final admission in April 2017, an ultrasound scan of the abdomen was performed which revealed a large amount of ascites, a nodular contour to the liver capsule and heterogeneous reflectivity within the liver in keeping with advanced cirrhosis. Prominent hepatic arterial branches were seen with a low vascular resistive index (0.62). Reversal of flow in the portal vein was demonstrated with a flow rate of 5 ml s^-1^ (Figure 4).

**Figure 5.**
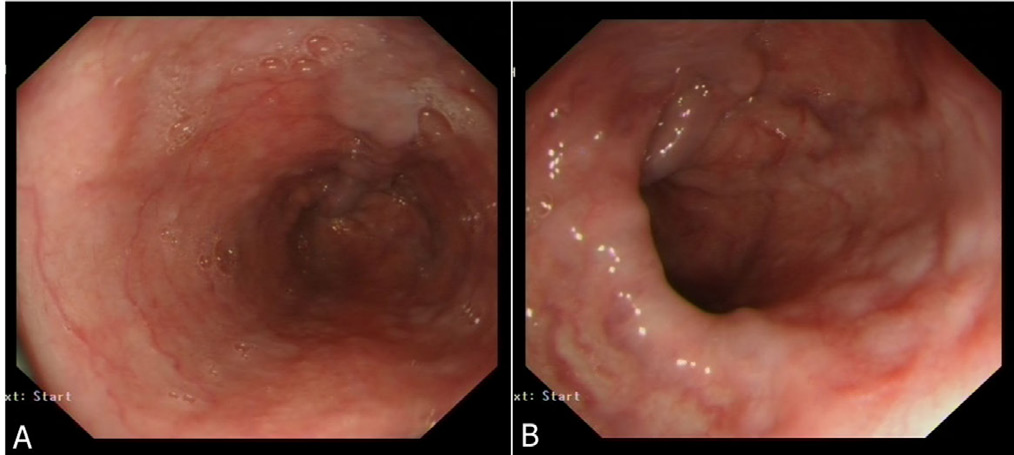
Oesophagogastroduodenoscopy late March 2017 demonstrating features consistent with portal vein hypertension and oesophageal varices.

Please note that an upper gastrointestinal endoscopy performed on 31 March 17 showed portal venous hypertensive changes and Grade II oesophageal varices, but no intraluminal mass (Figure 5A, B), based on which we would suggest that the gastrogastric intussusception seen in the earlier CT abdomen and pelvis study was intermittent.

## Case discussion

The case, which we have described above shares several demographic features with previously documented cases of gastrogastric intussusception. The patient’s age falls at the lower end of the literature review range of 65–83, but is still typical for both gastrogastric intussusceptions and intussusceptions generally in adults. The patient presented with similar non-specific clinical features as patients in previous case studies, namely abdominal pain, vomiting and general weakness, although it is felt more likely that these were attributable to the ultimate post-mortem diagnosis of spontaneous bacterial peritonitis secondary to liver failure and ascites.

One significant difference between the case of gastrogastric intussusception which we have described and those previously documented is the absence of a polypoidal lead point in our patient. This gentleman had no history of malignancy or other discrete abdominal soft tissue mass, and no evidence of such a lesion was seen on upper gastrointestinal endoscopy, radiological studies or following post mortem examination. We would like to suggest alternative pathophysiological mechanisms which may contribute to the development of a gastrogastric intussusception in the absence of a polypoidal lead point.

The patient had features of chronic liver disease with portal hypertension and oesophageal varices, as described above. Plackett et al have described a case of colocolonic intussusception occurring in a patient with a history of portal hypertension, ascites, retroperitoneal and peritoneal varices, and cirrhosis secondary to hepatitis C.^19^ This study has suggested that portal hypertension resulting in vascular congestion due to dilated and thickened capillaries may have functioned as a lead point for the development of colonic intussusception, and furthermore noted that this mural thickening became worsened as liver cirrhosis progressed. We postulate that the development of gastrogastric intussusception in our patient may have occurred due to a similar mechanism of vascular congestion on a background of portal hypertension, varices, and partial portal venous thrombosis, exacerbated by raised intra-abdominal pressure arising from ascites.

Hiatus hernia is a known risk factor for the development of intussusception in the absence of a polypoidal lead point: Ghahremani et al published a review of 12 patient cases involving oesophagogastric intussusception with concomitant sliding hiatus hernia. The main radiographic feature seen in these cases was a fundal mass surrounding the narrowed distal oesophagus and the mechanism for this distortion was suggested as being inversion of a hiatus hernia into the stomach.^20^ A similar case of retrograde gastro-oesophageal intussusception within a hiatal hernial sac was reported by El-Hajj et al with possible risk factors for the development of such a condition being deficiency in the gastric ligaments and omental attachments or the presence of a hiatus hernia with a lax phreno-oesophageal ligament.^21^

Whilst it is not known whether our patient exhibited any weaknesses in the fixation of his gastric ligaments, we would suggest that the presence of a hiatus hernia could still predispose individuals to gastrogastric intussusception via a similar mechanism, without simultaneous involvement of the oesophagus.

## Conclusions

Cases of gastrogastric intussusception are rarely reported in medical literature and patients presenting with this condition often have non-specific symptoms. We have described what we believe to be the first reported case of a gastrogastric intussusception occurring without a discrete gastrointestinal soft tissue lead point. We posit that portal venous hypertensive changes in the gastric wall, increased intra-abdominal pressure arising from the presence of ascites and the presence of a hiatus hernia could be alternative aetiological factors in the development of gastrogastric intussusception.

## Learning points

1. Gastrogastric intussusception is very rarely encountered in adults and is commonly associated with a polypoidal gastric tumour which can act as a lead point.
2. Cross-sectional imaging demonstrates typical findings of intussusception seen more familiarly elsewhere in the gastrointestinal tract, with low density intraluminal filling defects, target appearances and vascular pseudopedicles.
3. Portal hypertension and associated gastro-oesophageal varices, ascites, and hiatal hernia could predispose patients to gastrogastric intussusception in the absence of a solid lead point.
